# Synthesis, Structural and Biological Properties of the Ring-A Sulfonamido Substituted Chalcones: A Review

**DOI:** 10.3390/molecules26195923

**Published:** 2021-09-29

**Authors:** Malose J. Mphahlele

**Affiliations:** Department of Chemistry, College of Science, Engineering and Technology, University of South Africa, Private Bag X06, Florida 1710, South Africa; mphahmj@unisa.ac.za

**Keywords:** *o*/*m*/*p*-sulfonamidochalcones, molecular structure, biological activity, optical properties

## Abstract

Sulfonamidochalcones continue to assert themselves as versatile synthetic intermedi-ates and several articles continue to appear in literature describing their synthesis, chemical transformation and biological properties. These compounds are not only of interest from the medicinal chemistry context, their conformations and crystalline structures also continue to attract attention to explore non-covalent (intramolecular and intermolecular) interactions, control molecular conformations, and improve their physicochemical and optical properties. Despite an exhaustive list of examples of the ring-A sulfonamide-appended chalcones described in the literature, there is no com-prehensive review dedicated to their synthesis, structural and biological properties. This review focuses attention on the synthesis, structure and biological properties of the ring-A sulfonamide-appended chalcones (*o*/*m*/*p*-sulfonamidochalcones) as well as their potential as non-linear optical materials.

## 1. Introduction

**Figure 1:**
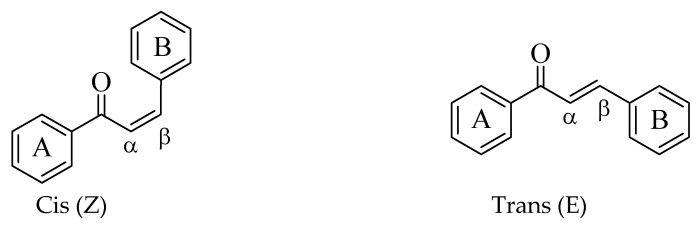
Generalized structure of chalcone isomers.

Recently, the concept of “one drug, multiple targets” has gained growing popularity and many researchers continue to synthesize and evaluate drug-like molecular constructs incorporating chalcone and sulfonamide moieties. Each of these pharmacophores possesses characteristic chemical reactivities and set of biological properties to serve as multifunctional drugs or multitarget-directed ligands. Chalcones (1,3-diphenylprop-2-ene-1-one) are considered as the precursors of flavonoids and isoflavonoids, which are abundant in edible plants and display a wide range of pharmacological activities [1]. Their bioactivity is correlated with the different electron-donor and electron-acceptor groups attached to the aromatic rings on either side of the α,β-unsaturated carbonyl (–CO–CH=CH-) framework [2]. The presence of this conjugated linkage between the two aromatic rings provides the cis (Z) and trans (E) isomers (Figure 1). The trans isomer is thermodynamically more stable and predominates over the cis conformer [3]. The latter is destabilized by steric interactions due to the proximity of ring A to the carbonyl group. Chalcones are ambident electrophiles with propensity to react with nucleophiles at the carbonyl group (1,2-addition) or the β-carbon (1,4- or Michael addition). Their α,β-unsaturated carbonyl moiety is involved in many biochemical signalling pathways in cells as a Michael acceptor [4]. The conjugated ketoethylenic scaffold and a completely delocalized π-electron system have also been found to reduce their redox potentials and make chalcones to be prone to electron transfer reactions [2]. There are a number of comprehensive reviews in the literature that focused predominantly on the pharmacological and chemical basis of the biological activities exhibited by hydroxychalcones with scant mention of their aminochalcone analogues. The latter are distinguished from the hydroxychalcones by the presence of an amino group attached to either ring A or B. These open chain flavonoid precursors have been extensively studied in recent decades as building blocks in combinatorial and diversity-oriented synthesis, because they are excellent 1,3-dielectrophilic systems.

**Figure 2:**
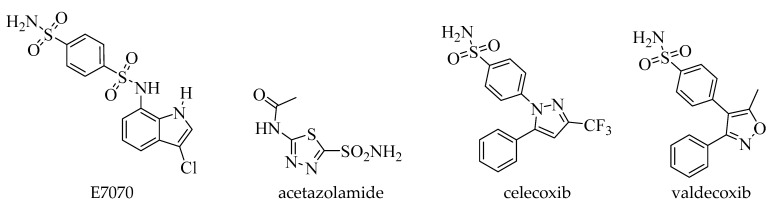
Structures of selected drugs containing sulfonamide group/s.

Sulfonamide-based compounds have one of the broadest ranges of biological activities among drug molecules and several derivatives are in the market. Some derivatives serve as oral diuretics (furosemide, indapamide, chlorthalidone, thiazides); anticancer (E7070), carbonic anhydrase (CA) inhibitors (acetazolamide, dichlorophenamide, dorzolamide and brinzolamide), antiepileptics (zonisamide and sulthiame), antiviral (darunavir), anti-inflammatory (celecoxib), antibacterial (sulfadizine), anticonvulsant (zonisamide) and cyclooxygenase-2 (COX-2) inhibitors (valdecoxib), while the others are used as ophthamologicals (dorzolamide) and for the treatment of rheumatoid arthritis (sulfasalazine) [5]. The structures of selected examples of these medicinally important drugs are included in Figure 2.

**Figure 3:**
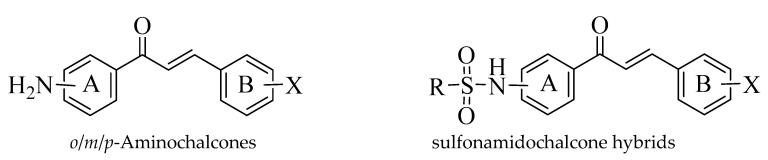
Generalized structure of the A-ring sulfonamido substituted chalcones.

Several authors have transformed the amine moiety of the *o*/*m*/*p*-aminochalcones (Figure 3) to comprise a wide range of sulfonamidochalcone hybrids with interesting biological properties and have also studied their geometry using spectroscopic and single crystal X-ray diffraction techniques complemented with computational methods. However, recourse to the literature revealed only one comprehensive review on the biological activities of the A- and B-ring substituted aminochalcones and their molecular hybrids, which included the sulfonamidochalcones of the generalized structure shown in Figure 3 [6]. Surprisingly, majority of the examples of the sulfonamidochalcones described in this comprehensive review on the aminochalcones are the *para*-sulfonamidochalcones and to a limited extend the *meta*-sulfonamidochalcones. Despite an exhaustive list of examples of the 2-aminochalcones and their biological properties described in this review, the corresponding data for the *ortho*-sulfonamidochalcone derivatives does not feature at all.

The *ortho*-sulfonamidochalcones are not only of interest from the medicinal chemistry context, their conformations and crystalline structures also continue to attract attention to explore intramolecular and intermolecular non-covalent interactions, control molecular conformations and improve the physicochemical properties [7,8,9,10,11,12,13,14,15,16,17]. Intramolecular hydrogen bonding has been found to result in conformational restriction of small drug molecules and, in turn, increased lipophilicity, membrane permeability and pharmacological activity [18]. As a result, it has become an exciting challenge in biological chemistry especially in the context of drug-receptor interactions to integrate intramolecular hydrogen bonding formation in drug design. This noncovalent interaction has been found to lead to favourable alignment of the drug-like molecules with the protein pocket resulting in increased ligand-receptor interactions [19]. Our long-standing interest in the chemistry and biological activity of aminochalcones and their derivatives prompted the need to provide a complementary comprehensive review to that of Irfan et al. [6] dedicated to the synthesis, biological activity and structural properties of the A-ring sulfonamido-appended chalcones reported to-date.

## 2. Synthesis of *o*/*m*/*p*-(Sulfonamido)Chalcones

**Scheme 1:**
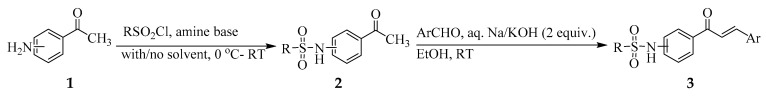
Claisen-Schmidt aldol condensation of sulfonamidoacetophenones (**2**).

A number of synthetic routes have been reported for synthesis of the *o*/*m*/*p*-(sulfonamido)chalcones and their general synthesis involves the Claisen-Schmidt aldol condensation of *o*/*m*/*p*-sulfonamidoacetophenone precursors with benzaldehyde derivatives in the presence of an acid [20] or base [2,7,8,9,10,11,12,13,21,22,23,24,25] followed by spontaneous in situ dehydration of the incipient β-hydroxyketone intermediates. The sulfonamidoacetophenones **2** are, in turn, prepared from the corresponsing *o*/*m*/*p*-aminoacetophenones **1** with alkyl/arylsulfonyl chloride derivatives in the presence of an amine as a base in the absence or presence of a solvent (Scheme 1). Treatment of **2** with benzaldehyde derivatives in the presence of a base or an acid catalyst in methanol or ethanol at room temperature (RT) or under reflux afford the corresponding *o*/*m*/*p*-sulfonamidochalcones **3**. This is the most commonly used efficient, high yielding and sure-fire approach to afford sulfonamidochalcones with variable substituents on the sulfonamide moiety (R = -CH_3_ or aryl) and the 3-aryl ring. In most cases, the compounds are isolated by aqueous work-up and purification by recrystallization from appropriate solvents.

**Scheme 2:**
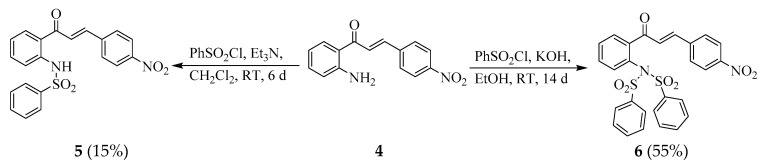
Reactions of **4** with phenylsulfonyl chloride to afford **5** and **6**.

Only few examples involving direct sulfonylation of aminochalcones have been reported in the literature. 2-Aminochalcone was previously treated with either methanesulfonyl chloride-pyridine mixture or benzenesulfonyl chloride-triethylamine mixture in dichloromethane at 0 °C to room temperature for 12–14 h to afford 2′-(methanesulphonamido)- and 2′-(benzenesulphonamido)chalcone in 19% and 25% yield, respectively [12]. Direct sulfonylation of (*E*)-1-(2-aminophenyl)-3-(4-nitrophenyl)prop-2-en-1-one **4** with benzenesulfonyl chloride in the presence of 50% potassium hydroxide as catalyst in ethanol at room temperature for 14 days previously afforded a benzenesulfonamidochalcone derivative **5** in 15% yield (Scheme 2) [11]. Treatment of this 2′-amino-4-nitrochalcone with benzenesulfonyl chloride in the presence of triethylamine as catalyst in dichloromethane at room temperature, on the other hand, afforded after 6 days an *N*-disulfonamide chalcone hybrid **6** in 55% yield (Scheme 2).

**Scheme 3:**
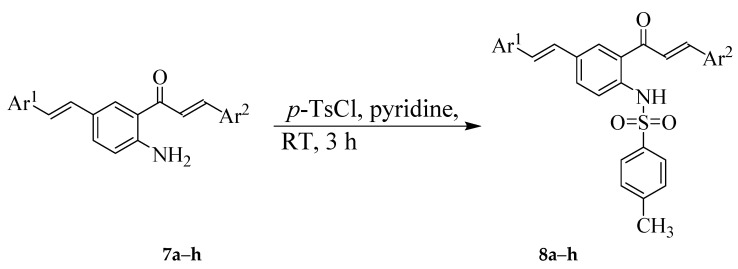
Sulfonylation of **7a**–**h** to afford the 5-styryl-2-sulfonamidochalcone hybrids **8a**–**h.**

A series of the 5-styryl–2-aminochalcone hybrids **7a**–**h** were previously subjected to 1.2 equiv. of *para*-toluenesulfonyl chloride (*p*-TsCl) in pyridine at room temperature for 3 h followed by aqueous work up and purification by silica gel chromatography to afford the corresponding 5-styryl-2-(tolylsulfonamido)chalcone hybrids **8a**–**h** in appreciable yields (Table 1, Scheme 3) [26]. No traces of the bisarylsulfonamide derivatives were detected or isolated from the reaction mixtures.

Sulfonamidochalcones are interesting synthetic intermediates as they provide a versatile platform for the synthesis of bioactive scaffolds such as cyanopyridines, isoxazoles, pyrazoles and pyrimidin-2-thiones [25]. The presence of the ambident electrophilic α,β-unsaturated carbonyl framework and its proximity to the nucleophilic *ortho*-sulfonamido group have long been exploited as templates for the base-mediated cyclization to afford the 2-aryl-1-(alkyl/arylsufonyl)-2,3-dihydroquinolin-4(1*H*)-ones [27,28,29] with increased propensity to undergo regio- and stereoselective C-3 halogenation [30]. Base-mediated cyclization-condensation with benzaldehyde derivatives, on the other hand, afforded the 2-aryl-1-(arylsulfonyl)-3-benzylidene-2,3-dihydroquinolin-4(1*H*)-ones [31,32].

## 3. Structural Properties of *o*/*m*/*p*-(Sulfonamido)Chalcone Derivatives

The solid-state structures and geometries of *o*/*m*/*p*-sulfonamidochalcones have been extensively studied using single crystal X-ray diffraction (XRD) complemented with the Hirshfeld surface analysis to give more accurate description of the intermolecular interactions. Nuclear magnetic resonance (NMR) spectroscopic techniques have also been exploited to study the structures and geometries of chalcones in the solution phase. Moreover, quantum chemical techniques have also been employed to simulate the structures and energies of sulfonamidochalcone in the gas and solution phases to complement spectroscopic and/or X-ray data. These analytical techniques revealed that the conjugated carbon scaffolds of *o*/*m*/*p*-aminochalcones and their sulfonamide derivatives are essentially planar. However, twisting of the sulfonamide moiety from coplanarity of the conjugated carbon framework is commonly observed in the crystal structures of the *ortho*- [7,8,9,10,11,12,13], *meta*- [22,23] and *para*-sulfonamido appended chalcones [24,33]. The twisting of the sulfonamide moiety from coplanarity of the conjugated carbon framework has also been observed in the crystal structures of the analogous *N*-(2-acetyl-4-(styryl)phenyl)sulfonamides [34] and the corresponding 5-styryl-2-sulfonamidochalcone hybrids [26]. The distorted tetrahedral geometry of the sulfonamide moiety, on the other hand, makes its oxygen atoms to engage in several hydrogen bonding interactions [17]. As a result, this moiety represents a versatile template to explore hydrogen bonding interactions, control molecular conformations and improve the physicochemical properties of the drug molecules [16,17]. The Hirshfeld surface analyses and molecular potential maps of sulfonamidochalcones confirm the preponderance of intermolecular interactions of the type C–H···O and N–H···O which stabilize the crystal packing. Aromatic-aromatic (π···π) stacking and C–H···π interactions have also been observed in some cases. The study of these non-covalent interactions is crucial to extrapolate structure activity relationship (SAR) and also play a significant role in layered materials [8]. Crystal packing of the sulfonamidochalcones is generally stabilized by intermolecular hydrogen bonding and additionally by a pseudo-ring formed through intramolecular hydrogen bonding interaction between the carbonyl oxygen and NH in the case of the *ortho*-sulfonamido analogues. Besides the classical hydrogen bonds, other different kinds of supramolecular interactions such as H···H, CH···O and C···O are also important for the stabilization of the crystal structures of sulfonamidochalcones.

**Figure 4:**
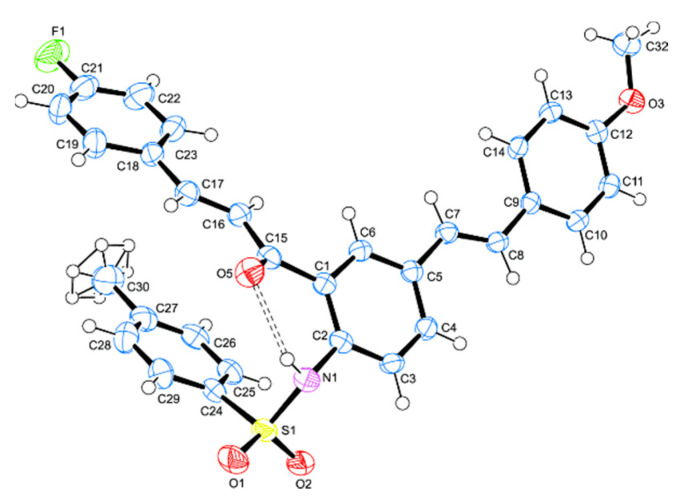
ORTEP diagram of compound **8h** with thermal ellipsoids drawn at 50% probability. CIF file of this compound (CCDC 2043901) is freely available from the following website: www.ccdc.cam.ac.uk/datarequest/cif. (accessed on 26 August 2021).

XRD analyses of the 2-aminochalcones and their *ortho*-sulfonamidochalcone derivatives confirm the presence of a six-membered N–H···O intramolecularly hydrogen bonded ring in co-planarity with the conjugated scaffold [7,8,9,10,11,12,13]. The highest frequency of intramolecular hydrogen bonds for the 2-aminochalcone derivatives found in the literature have planar six-membered pseudo ring stabilized by conjugation with a π-system, which mimic an aromatic ring [35]. A thermodynamically favoured chair-like conformation was, however, observed for the first time in the crystal structure of 5-styryl-2-(tolylsulfonamido)chalcone hybrid **8h** (Figure 4) due to the twisting of the tolyl group about the trigonal nitrogen atom towards the chalcone arm [26]. This alignment caused the carbonyl oxygen atom of the chalcone wing to deviate from co-planarity resulting in a chair-like conformation of the pseudo ring.

NMR spectroscopy has also been used extensively to study the structure and geometry of chalcone derivatives. Increased number of signals in the aromatic region (δ 6.0–7.5 ppm) of their ^1^H NMR spectra of chalcone derivatives serves to confirm the successful condensation between acetophenone and benzaldehyde. A notable feature in the ^1^H NMR spectra of chalcones is the presence of two sets of doublets among the aromatic proton signals with vicinal coupling constant (*J*_vic_) values of 15.0–16.5 Hz characteristic of the *trans* geometry of the enone framework. Their ^13^C NMR spectra also show increased resonances compared to the corresponding precursors with distinct signals for the C=O, >C–H and =C– fragments. The singlet for the carbonyl carbon resonates around δ = 191.0 ppm in their ^13^C-NMR spectra confirming their α,β-unsaturated carbonyl nature. The exchangeable secondary amide (-SO_2_NH) proton in the case of the m/p-sulfonamidochalcones resonates as a broad singlet significantly downfield in their ^1^H NMR spectra around δ 10.8 ppm due to the deshielding effect of the S=O bonds. This singlet in the case of the *o*-sulfonamidochalcones resonates as a relatively sharp singlet around δ 11.5 ppm and this significant downfield shift is also consistent with participation of NH in intramolecular hydrogen bonding interaction with the carbonyl oxygen. The difference in line widths of this signal for the *m*/*p*-sulfonamidochalcones versus the *ortho* isomers is due to H/D exchange with the solvent molecule. The infrared (IR) spectra of sulfonamidochalcones reveal the presence of strong absorption bands in the regions 1295–1392 cm^−1^ and 1117–1185 cm^−1^ which correspond to the SO_2_ modes due to the asymmetric and symmetric S=O stretching vibrations, respectively [12,36]. Several research groups have also studied the biological activity of sulfonamidochalcones against various biological and molecular targets which are summarized below.

## 4. Biological Activities of Sulfonamidochalcones

An exhaustive list of examples of the *p*-sulfonamidochalcones and their biological properties as anticancer, enzyme inhibitors and antiparasitic agents have been discussed in the review by Irfan et al. [6]. The biological activities of some of the examples of the *p*-sulfonamidochalcones not included in that review are briefly discussed below. The main focus below is on the biological activity of the *m*-sulfonamidochalcones and the *o*-sulfonamidochalcones which have been sparsely or not described in this comprehensive review on the biological activities of aminochalcone derivatives.

### 4.1. Sulfonamidochalcones as Enzyme Inhibitors

**Figure 5:**
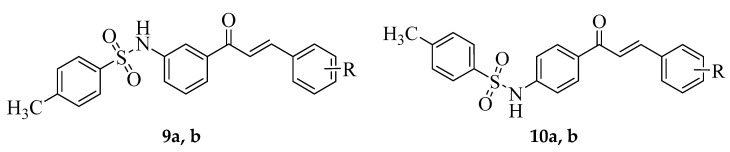
Sulfonamidochalcones with antihyperglycaemic properties.

The propensity of the sulfonamide moiety to engage in electrostatic and noncovalent bonding interactions with protein residues in the receptor binding site, affords sulfonamide-based compounds their ability to inhibit various enzymes. Several examples of sulfonamidochalcones have previously been evaluated for inhibitory effect against enzymes such as tyrosinase [33], α-glucosidase [26,37], α-amylase [26,37], β-amylase [37], β-secretase and acetylcholinesterase [38]. The inhibitory effect of the *m*-sulfonamidochalcones of the generalised structure **9** (Figure 5) and their *m*-aminochalcone precursors were previously evaluated in vitro against α-amylase, β-amylase and α-glycosidase [37]. The *m*-aminochalcone precursors were generally inactive against the three enzymes. The activity of the *m*-sulfonamidochalcones **9a** and **9b** was found to be stronger against all three enzymes, and more so against the amylases (Table 2). It was concluded that the difference in inhibitory effect against these enzymes was due to the presence of sulfonamide group, which favoured the inhibition of α- and β-amylases than α-glucosidase. The *para*-sulfonamido isomers **10a** and **10b**, on the other hand, exhibited stronger inhibitory effect against α-glucosidase and modest activity against both amylases.

The biological activities of the 5-styryl-2-(tolylsulfonamido)chalcone hybrids **8a**–**h** as potential anti-diabetic agents have been evaluated in vitro through enzymatic assays against α-glucosidase and α-amylase activities [26]. The presence of a sulfonamido group on the 5′-(3-fluorostyryl)-chalcone scaffold of **8a**–**d** resulted in moderate to significant inhibitory effect against α-glucosidase compared to acarbose (IC_50_ = 0.93 ± 0.15µM) used as a reference stndard for the assay (Table 3). The activity of these compounds against α-glucosidase was found to decrease with the increasing size of the substituent on the *para* position of ring-B, and the trend was as follows, **8a** > **8b** > **8c** > **8d**. The inhibitory effect of compounds **8a**–**d** against this enzyme was found to be higher than that of the corresponding substrates **7a**–**d** observed in the preceding study [39]. These compounds were found to be moderately inhibiting against α-glucosidase compared to the reference drug, acarbose (IC_50_ = 0.93 ± 0.15 µM), with IC_50_ values in the range 3.2–12.5 µM (Table 3). The 5′-(4-fluorostyryl) substituted derivative **8e** was the lesat active within the series **8e**–**h** with an IC_50_ value of 12.5 ± 0.42 µM. Its activity against α-glucosidase was found to be significantly lower than that of its isomer **8a** (IC_50_ = 4.2 ± 0.21 µM) and the corresponding substrate **7e** (IC_50_ = 5.1 ± 0.61 µM). Significant inhibitory effect agaisnt α-glucosidase was observed for **8f** (IC_50_ = 3.2 ± 0.33 µM) substituted with the 4-fluorophenyl group on the styryl and chalcone arms. The activity of this compound was found to be higher than that of the isomer **8b** (IC_50_ = 5.4 ± 0.10 µM), and twice higher than that previously observed for the corresponding substrate **7b** (IC_50_ = 6.9 ± 0.37 µM) [39]. The docking pose of compound **8h** revealed π-π stacking interactions with the side chain of α-glucosidase, and the hydrogen bond is envisaged to be mainly contributed by its carbonyl group. The presence of sulfonamide group at the ortho [39], *meta* or *para* position of ring A [40] generally resulted in increased inhibitory effect against α-glucosidase compared to the corresponding aminochalcone precursors. The 5-styryl-2-(tolylsulfonamido)chalcone hybrids **8a**–**h** were also evaluated for inhibitory effect in vitro against α-amylase (Table 3) [26]. Replacement of the amino group of **7a**–**d** with a sulfonamido group resulted in variable activity for derivatives **8a**–**d** against α-amylase with IC_50_ values in the range 4.8 ± 0.25–13.6 ± 0.15 µM. A combination of the 5-(3-fluorostyryl) and 4-chlorophenyl group on the chalcone wing of compound **8c** resulted in significantly reuced inhibitory effect agaisnt this enzyme compared to substrate **7c** [39] with IC_50_ values of 13.6 ± 0.15 μM and 2.4 ± 0.10 μM, respectively. The other derivataves within this series exhibited comparable activity against α-glucosidase and α-amylase. Both **8e** and **8f** exhibited reduced inhibitory effect against α-amylase compared to the isomeric **8a** and **8b**. The presence of the sulfonamido group in **8e** also resulted in significantly reduced activity against α-amylase compared to substrate **7e** (IC_50_ = 1.6 ± 0.52 μM [39]) and the corresponding IC_50_ value is 12.5 ± 0.18 μM. The 5-(4-chlorostyryl) **8g** (IC_50_ = 4.1 ± 0.61 μM) and the 5-(4-methoxystyryl) **8h** (IC_50_ = 2.1 ± 0.53 μM) substituted derivatives exhibited significant activity against α-amylase than the corresponding isomers **8c** and **8d**, respectively. Hitherto, the IC_50_ values for the corresponding substrates **7g** and **7h** were found to be 1.7 ± 0.25 μM and 7.6 ± 0.20 μM, respectively [39]. The docking pose of **8h** in the active site of α-amylase revealed Leu165 and Ile235 as the two command residues that made hydrophobic interactions with this compound [26].

### 4.2. Sulfonamidochalcones as Anticancer Agents

**Figure 6:**
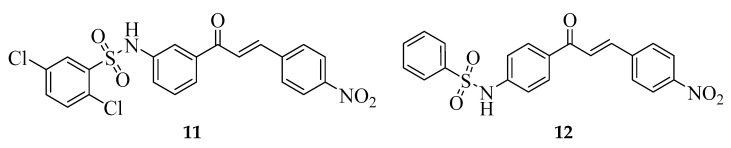
Examples of *m*/*p*-sulfonamoido-3-(4-nitrophenyl)chalcones with biological activity.

The antitumor effect of sulfonamidochalcones has been evaluated in vitro on several cancer cell lines including the human liver cancer (HEPG2) [41], breast cancer (MCF-7) [42], glioblastoma (SF-295), prostate cancer (PC-3) and colorectal cancer (HCT-116) [22] cells. Cytotoxicity analysis of 2,5-dichloro-*N*-{3-[(2*E*)-3-(4-nitrophenyl)prop-2-enoyl]phenyl}benzenesulfonamide (**11**) shown in Figure 6 was performed using the tetrazolium 3-(4,5-dimethyl-2-thiazole)-2,5-diphenyl-2-*H*-bromide salt (MTT) assay against the human glioblastoma (SF-295), prostate cancer (PC-3) and colon cancer (HCT-116) cell lines [22]. Based on the docking scores of −16.08 and −17.45 against nuclear retinoic acid receptors alpha (RARa) and beta (RARb), it was suggested that this sulfonamidochalcone act as an anticancer agent by interacting with these receptors [22]. A combination of sulfonamide and chalcone moieties in *N*-(4-[3-(4-nitrophenyl)prop-2-enoyl]phenyl)benzenesulfonamide (**12**) resulted in significant cytotoxicity in vitro against the human liver cancer (HEPG2) compared to the corresponding precursor, 4-acetyl-*N*-(*p*- tolyl)benzenesulfonamide (IC_50_ = 35.8 µM), and the reference drug doxorubicin (IC_50_ = 3.3 µM) with an IC_50_ value of 26.0 µM [41]. Reduced cytotoxicity was observed for the corresponding oxime, pyrazole and pyrimidine derivatives, which confirmed the importance of the α,β-unsaturated ketone system in chalcones as an anticancer pharmacophore. The genotoxic, cytotoxic, antigenotoxic, and anticytotoxic activities of **12** have also been assessed using the *Salmonella typhimurium* reverse mutation test (Ames test) and the mouse bone marrow micronucleus test [43]. The genotoxic and cytotoxic actions of CPN were attributed to the inhibition of tubulin formation through hydrophobic interaction between the oxygen atom of the sulfonamide moiety and the amino hydrogen present in tubulin lysine residues.

**Figure 7:**
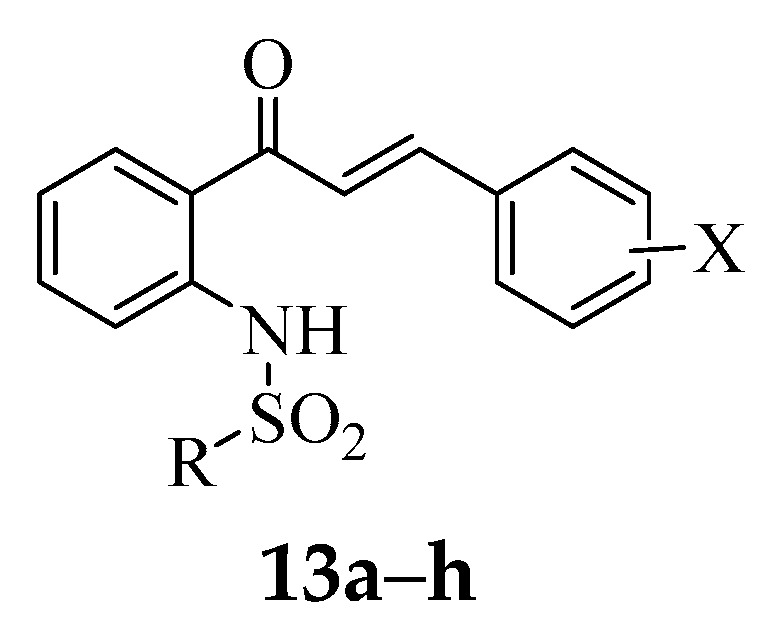
*Ortho*-sulfonamidochalcones with cytotoxicity against SF-295 and PC-3.

A series of *ortho*-sulfonamidochalcones **13** (Figure 7) were evaluated for cytotoxicity against tumor cell lines SF-295 and PC-3 [10]. The tested compounds showed IC_50_ values ranging from 2.1 to 7.9 mg mL^−1^ against the two cancer cell lines (Table 4). Hitherto, these compounds exhibited significant cytotoxicity against the HCT-116 cell line with IC_50_ values ranging from 2.4 to 7.5 µg mL^−1^ [44]. Compounds **13a**–**h** were, in turn, docked into the active site of the mitogen activated protein kinase 10 (JKN3, PDB code: 1PMV), which is known to be inhibited through intermolecular interactions involving both GLU-147 and MET-149 residues [10]. Molecular docking suggested that these *ortho*-sulfonamidochalcones inhibit JNK3 by binding to alternative residues than the specific JKN3 inhibitor, dihydroanthrapyrazole (ATRP). The inhibitory activity of the most active derivative **13g** was predicted to be governed by the interactions between GLN-75 and the chlorine atom (2.7 A and 3.6 A) with better binding affinity to JNK3 (−10.0 kcal mol^−1^) than ATRP (−9.1 kcal mol^−1^). It was concluded that chlorine atom in the *para*-position of the 3-phenyl ring plays a key role in its biological potential. The *ortho*-sulfonamidochalcones and their quinolinone-chalcone hybrids were also evaluated for cytotoxicity against PC-3 (prostate), HCT-116 (colon) and SF-295 (glioblastoma) human tumor cell lines [13]. Non-enzyme catalyzed nucleophilic addition of reduced glutathione (GSH) onto two of these sulfonamidochalcones and their corresponding quinolinone-chalcone hybrids were also studied to investigate the relationship between tumor cell cytotoxic activities and GSH-reactivities of the compounds. The nonplanar quinolinone-chalcone hybrids exhibited reduced thiol-reactivity due to fast decomposition of their GSH-conjugates. A combined X-ray diffraction and theoretical approach confirmed that the coplanarity of the conjugated carbon framework of the *ortho*-sulfonamidochalcones due to intramolecular hydrogen bonded favoured formation of the respective GSH-conjugates.

The 5-styryl-2-(sulfonamido)chalcones **8f** and **8h** (refer to Scheme 3 above) which exhibited inhbitory activity against both carbohydrate hydrolyzing enzymes (α-glucosidase and α-amylase) as well as free radical scavenging proprties were also evaluated for cytotoxicity against the A549 cell line and the normal monkey kidney cells (Vero cells) [26]. These compounds exhibited reduced cytotoxicity against the A549 cell line (IC_50_ = 52.40 ± 0.09 µM and 50.30 ± 0.11 µM, respectively) and did not affect the viability of the Vero cells (IC_50_ = 120.90 ± 0.67 µM and 168.60 ± 0.16 µM, respectively) compared to doxorubicin (IC_50_ = 0.66 ± 0.12 µM and 1.14 ± 0.23 µM, respectively) used as a reference standard for the assay. It is envisaged that further cellular-based in vitro and in vivo studies including bioavailability and cell permeability would help clarify the mechanism of action of these compounds in the body, and to establish their safety profile as potential multi-target agents against the pathogenesis and progression of T2DM.

### 4.3. Sulfonamidochalcones as Anti-Inflammatory Agents

**Figure 8:**
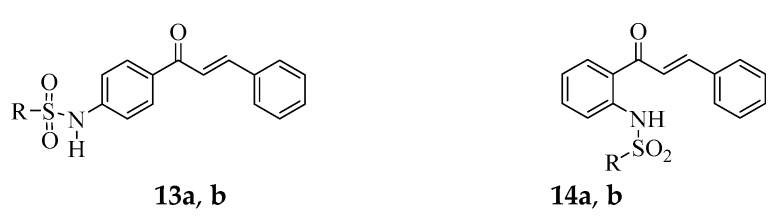
Structures of **13a**, **b** and **14a**, **b**.

The in vitro anti-inflammatory potential of the ring-A monosubstituted chalcone derivatives was previously evaluated against denaturation of bovin serum albumin (BSA) in the presence of ibuprofen as a reference drug [12]. It was found that the substitution pattern on the phenyl ring of the acetophenic group of chalcone moiety plays an important role in modulating inflammation. The observed trend in anti-inflammatory activity for the primary amino A-ring substituted chalcones was as follows: *ortho* > *para* > *meta*. The preference for the *ortho* position is due to the superior ability of the amino group to form an intramolecular hydrogen bond with the carbonyl oxygen. The *p*-sulfonamido chalcones **13a** and **13b**, and the *ortho*-substituted isomers **14a** and **14b** shown in Figure 8 were found to be the most active derivatives across the concentration range (10, 20 and 30 µg mL^−1^) tested (Table 5). The observed increase in the percent inhibition of the sulfonamidochalcones over the parent chalcones and the reference standard underscores the importance of this functional group as a ring A substituent. Increased activity in the case of the benzenesulphonamide substituted derivative **14b** was attributed to the presence of the phenyl group of the sulfonamide moiety which is likely participate in hydrophobic interactions with the relevant residues in the cyclooxygenase-2 (COX-2) binding pocket similar to those established in the case of a *p*-NHSO_2_CH_3_ group.

### 4.4. Sulfonamidochalcones as Antioxidants

**Figure 9:**
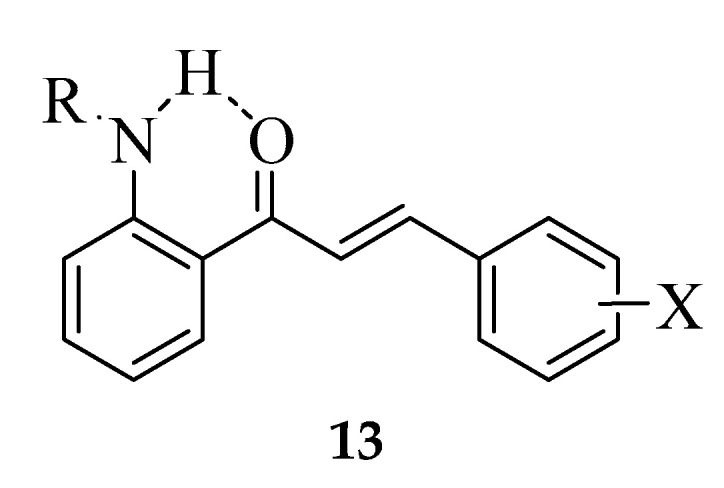
*o*-Amino substituted chalcone scaffold showing N-H···O hydrogen bonded pseudo ring.

The *p*-sulfonamido chalcones **13a** and **13b**, and the *ortho*-substituted isomers **14a** and **14b** were also evaluated for antioxidant activity through the 2,2-diphenyl-1-picrylhydrazyl (DPPH) radical scavenging assay using ascobic acid as a reference standard [12]. Compounds **13a**, **13b**, **14a** and **14b** exhibited reduced activity compared to ascorbic acid (% inhibition = 96.43 ± 0.21) at the highest concentration tested (50 µg mL^−1^) with lower % inhibition values of 11.34 ± 0.44, 11.55 ± 0.14 and 7.84 ± 0.28, respectively. Reduced or lack of antioxidant activity of the 2-aminochalcones and their sufonamido derivatives was attributed to the presence of intramolecular N-H···O hydrogen bond between the NH and the carbonyl oxygen [35,43]. This pseudo ring depicted in structure **13** represented in Figure 9 is envisaged to deactivate the unsaturated moiety resulting in reduced free radical scavenging activity of the *ortho*-aminochalcone derivatives. The 2-aminochalcones with different substitution pattern on the B-ring and the 4-aminochalcones and the 4-(sulfonamido)-substituted chalcones were previously evaluated for antioxidant activities by the DPPH radical scavenging method and also found to be inactive [45].

Compounds **8a**–**h** shown in Scheme 3 above which incorporate the styryl, the electrophilic α,β-unsaturated carbonyl arm and the sulfonamide moiety on the same molecular framework were also evaluated for antioxidant properties in vitro through the DPPH and nitric oxide (NO) radical scavenging assays [26]. Compounds **8c**, **8d**, **8e** and **8g** exhibited significant DPPH radical scavenging activity with IC_50_ values of 8.3 ± 0.53 μM, 5.1 ± 0.23 μM, 7.9 ± 0.48 μM and 8.5 ± 0.38 μM, respectively, compared to ascorbic acid (IC_50_ = 4.57 ± 0.21 μM). Compounds **8d** (IC_50_ = 8.4 ± 0.61 μM) and **8e** (IC_50_ = 7.3 ± 0.54 μM) also exhibited significant antioxidant effect in the NO assay against ascorbic acid (IC_50_ = 6.33 ± 0.14 μM). The observed free radical scavenging activities of these molecular hybrids was attributed to the presence of styryl arm on the intramolecularly hydrogen bonded 2-sulfonamidochalcone scaffold. The antioxidant activity of the (E)-stilbenes such as the naturally occurring resveratrol (*trans*-3,4,5-trihydroxystilbene) and pterostilbene (trans-3,5-dimethoxy-4-hydroxystilbene) and their analogues has been found to be influenced by the electron-donating or electron-withdrawing nature of the functional groups at the 4 and 4’ positions, respectively [46]. Compounds, **8f** and **8h** have also been subjected to an in vitro cell-based antioxidant activity assay involving lipopolysaccharide (LPS) induced reactive oxygen species (ROS) production, which also confirmed their capability to scavenge free radicals [26]. Molecular modelling of **8h** confirmed that the free radical attack is favoured at the carbonyl group (f° = 0.0563) and carbon *ipso* to the styryl wing (f°= 0.0414), β-carbon of the styryl wing (f° = 0.0401), at the β-carbon (f° = 0.0372) of the chalcone arm, and also at the carbon atoms *meta* (f° = 0.0328) and *ipso* (f° = 0.0315) to sulfonamide group, respectively [26].

### 4.5. Sulfonamidochalcones as Antimicrobial Agents

**Figure 10:**
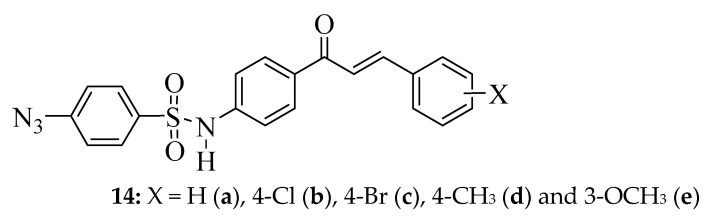
Azidosulfonamidochalcone derivatives **14** evaluated for antimicrobial properties.

Azidosulfonamidochalcone derivatives of the generalised structure **14** (Figure 10) were synthesized and tested for their antimicrobial activity against a wide variety of Gram-positive, Gram-negative, and fungal strains [47]. Three azidosulfonamidochalcones **14a** (X = H), **14b** (X = 4-Cl) and **14c** (X= 4-Br) showed relatively broad activity against the tested strains compared to 4-azido-*N*-(4-acetylphenyl)benzenesulfonamide used as a substrate for their preparation. A combination of the sulfonamido and chalcone scaffolds in compounds 14 resulted in increased antibacterial activity for the phenyl **14a** (X = H) and 4-chloro **14b** (X = Cl) substituted sulfonamidochalcones toward *Staphylococcus aureus*, *Micrococcus luteus*, and *Serratia marcens* compared to ampicillin trihydrate used as a reference standard for the assays. All of the azidosulfonamidochalcones exhibited moderate activity against *Klebsiella pneumonia* and a lower ability to inhibit *Escherichia coli* growth. Among the six fungal species tested, the most potent derivatives 4-halogeno substituted derivatives **14b** (X = 4-Cl) and **14c** (X = 4-Br) demonstrated strong activity toward the fungal strains, *Trichophyton rubrum* and *Geotrichum candidum*. In silico screening of these derivatives showed hydrophobic interactions of the chalcone framework and the sulfonamido group with several protein residues (Arg68, Pro69, Arg254, Lys220, Gly188, Ser221, and Pro193) in the dihydropteroate synthase (DHPS) binding site. Interactions of the docked sulfonamidochalcone derivatives indicated their substantial ability to occupy the pocket preventing the substrate STZ-DHPP from binding, leading to the inhibitory activity of the prepared compounds against DHPS. 

### 4.6. Sulfonamidochalcones as Antiparasitic Agents

**Figure 11:**
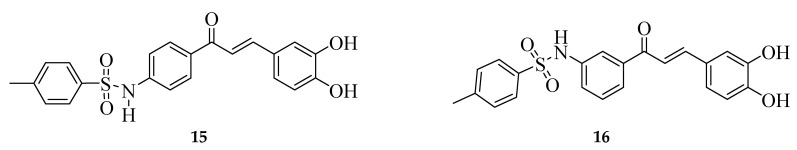
*p*-Sulfonamido- and m-sulfonamidochalcones derived from 3,4-dihydroxybenzaldehyde.

The *o,m,p*-(tolylsulfonamido)-containing hydroxylated chalcone derivatives were synthesised and evaluated for inhibitory effect against the trans-sialidase from *Trypanosoma cruzi* (TcTS) [20]. Increased anti-leishmanial activity was observed for the *p*-sulfonamidochalcone **15** and *m*-sulfonamidochalcone **16** derived from 3,4-dihydroxybenzaldehyde with IC_50_ values of 0.9 µM and 2.5 µM, respectively (Figure 11).

**Figure 12:**
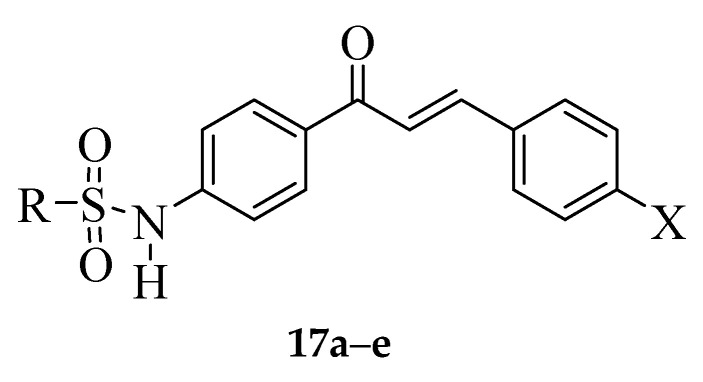
*p*-Sulfonamidochalcones previously evaluated for antifilarial properties.

A series of sulfonamidochalcones (27 examples) synthesized from *p*-aminoacetophenone and benzenesulfonyl chloride, methanesulfonyl chloride or tolylsulfonyl chloride were evaluated in vitro through antifilarial assay against human lymphatic filarial parasite *Brugia malayi* [24]. Thirteen of the sulfonamidochalcones were found to exhibit significant antifilarial activity and to be therapeutically safe. Among them, derivatives **17a**–**e** (Figure 12) were found to have remarkable antifilarial activities with lower IC_50_ values compared to the other derivatives (Table 6). Furthermore these compounds showed evidence of possible apoptosis of the parasite by differential fluorescent staining suggesting drug induced apoptosis to be the primary reason for antiparasitic effect of these compounds.

### 4.7. Sulfonamidochalcones as Potential Nonlinear Optical Materials

Benzenesulfonamide derivatives exhibit good nonlinear optical (NLO) properties [8]. The delocalized electronic charge distribution and p-orbitals of chalcones, on the other hand, lead to a high electron density mobility [48]. A combination of these moieties on the same molecular framework makes sulfonamidochalcones ideal candidates for NLO materials. The linear and nonlinear properties of the *ortho-s*ulfonamidochalcones have been studied in the solid state and solution phase before, and it was found that the optical properties are less influenced in solution [7]. The nonlinear optical properties of the *ortho*-benzenesulfonamide appended chalcones with different substituents at the *para* position of ring B were found to compare favourably with those of the parent 2-aminochalcones [8]. It was concluded that the sulfonamide group does not modify the nonlinear optical properties as these compounds.

## 5. Conclusions

This review provides an up-to-date record on the chemistry, physicochemical and biological properties of the A-ring sulfonamide-appended chalcones. These compounds are not only of interest from the medicinal chemistry context, their conformations and crystalline structures also continue to attract attention to explore non-covalent (intermolecular and intramolecular) interactions. The proximity of the nucleophilic sulfonamido group to the ambident electrophilic α,β-unsaturated carbonyl moiety makes the *ortho*-sulfonamidochalcones important intermediates for further structural modification to afford novel biologically relevant heterocycles. The presence of halogen atoms on the A or B ring will facilitate transition metal mediated cross-coupling reactions to afford polycarbosubstituted chalcones and their heterocyclic derivatives. Structural modifications on the A and/or B rings as well as varying substituents on the sulfonamido group could afford molecular hybrids for application in medicine and materials will be designed and prepared in the future. The sulfonamidochalcones could also be complexed with transition metal to afford ligands with interesting biological properties. Further cellular-based in vitro and in vivo studies including bioavailability and cell permeability would probably help clarify the mechanism of action of sulfonamidochalcones in the body, and to establish their safety profile as potential multi-target agents against the pathogenesis and progression of several human disorders.

## Figures and Tables

**Table 1:** Designation and substitution of aryl groups for **8a**–**h.**

8a–h	Ar^1^	Ar^2^	%Yield
**8a**	C_6_H_5_-	3-FC_6_H_4_-	80
**8b**	4-FC_6_H_4_-	3-FC_6_H_4_-	74
**8c**	4-ClC_6_H_4_-	3-FC_6_H_4_-	70
**8d**	4-MeOC_6_H_4_-	3-FC_6_H_4_-	66
**8e**	C_6_H_5_-	4-FC_6_H_4_-	71
**8f**	4-FC_6_H_4_-	4-FC_6_H_4_-	68
**8g**	4-ClC_6_H_4_-	4-FC_6_H_4_-	68
**8h**	4-MeOC_6_H_4_-	4-FC_6_H_4_-	60

**Table 2:** Deisgnation of R and IC_50_ values of **9a**, **b** and **10a**, **b**.

Compounds	R	IC_50_ in µM		
		α-Amylase	β-Amylase	α-Glucosidase
**9a**	4-OH	37.3	201.4	12.4
**9b**	3,4-OH	16.8	24.8	15.6
**10c**	4-OH	87.8	247.3	0.98
**10d**	3,4-OH	193.7	65.0	0.40

**Table 3:** α-Glucosidase and α-amylase inhibition of compounds **8a**–**h.**

Compound	[IC_50_ (SD) µM]	
	α-Glucosidase	α-Amylase
**8a**	4.2 ± 0.21	5.1 ± 0.30
**8b**	5.4 ± 0.10	4.8 ± 0.25
**8c**	6.1 ± 0.56	13.6 ± 0.15
**8d**	8.1 ± 0.61	8.3 ± 0.40
**8e**	12.5 ± 0.42	12.5 ± 0.18
**8f**	3.2 ± 0.33	7.6 ± 0.54
**8g**	9.7 ± 0.65	4.1 ± 0.61
**8h**	5.9 ± 0.20	2.1 ± 0.53
Acarbose	0.93 ± 0.15	-
α-Amylase Inhibitor	-	0.39 ± 0.10

**Table 4:** Designation and position of substituent X and IC_50_ values of **13a**–**h.**

Compounds	X	IC_50_ in µg·mL^−1^ (95% Confidence Intervals)	
		SF-295	PC-3
**13a**	2-F	4.6 (4.0–5.3)	5.2 (4.8–5.6)
**13b**	2-Cl	4.0 (3.1–5.1)	5.1 (4.8–5.4)
**13c**	2-Br	7.6 (6.5–8.9)	7.5 (6.8–8.3)
**13d**	2-OMe	7.1 (5.8–8.7)	6.9 (6.2–7.7)
**13e**	3-NO_2_	7.9 (6.5–9.7)	6.6 (5.7–7.5)
**13f**	4-F	6.2 (4.3–8.9)	5.9 (5.2–6.7)
**13g**	4-Cl	2.1 (1.7–2.7)	2.4 (2.1–2.6)
**13h**	4-Br	5.0 (4.2–6.0)	5.9 (5.2–6.9)
Doxorubicin	-	0.4 (0.3–0.5)	0.1 (0.1–0.2)

**Table 5:** Designation of R and % inhibition of **13** and **14** against BSA.

Compound	R	% Inhibition		
		10 (µg mL^−1^)	20 (µg mL^−1^)	30 (µg mL^−1^)
**13a**	CH_3_-	85.11 ± 0.18	87.15 ± 0.18	88.76 ± 0.09
**13b**	C_6_H_5_-	84.15 ± 0.43	85.26 ± 0.04	86.04 ± 0.20
**14a**	CH_3_-	84.94 ± 0.33	86.69 ± 0.79	88.65 ± 0.24
**14b**	C_6_H_5_-	85.81 ± 048	87.95 ± 0.24	91.07 ± 0.09
Ibuprofen	-	80.37 ± 0.51	80.79 ± 0.41	84.29 ± 0.72

**Table 6:** Designation of X and R for compounds **17a**–**e** and their IC_50_ values.

17	X	R	IC_50_ (µM)
**17a**	F	C_6_H_5_-	4.5
**17b**	NO_2_	CH_3_-	4.4
**17c**	H	4-CH_3_C_6_H_4_-	9.7
**17d**	Br	4-CH_3_C_6_H_4_-	4.6
**17e**	CH_3_-	4-CH_3_C_6_H_4_-	8.2
